# Early outcomes of anterior segment parameters after implantable collamer lens V4c implantation

**DOI:** 10.1186/s12886-022-02656-9

**Published:** 2022-11-10

**Authors:** Qinghong Lin, Dong Yang, Xingtao Zhou

**Affiliations:** 1 Department of Ophthalmology, Eye and Ear, Nose, and Throat Hospital of Fudan University, No. 83 Fengyang Road, Xuhui District, Shanghai, 200000 China; 2 Refractive Surgery Department, Bright Eye Hospital, Shanghai, 200000 China

**Keywords:** Implantable collamer lens V4c, Anterior segment parameters, Myopia, Complications, Post-operative outcomes

## Abstract

**Background:**

This study investigated the early outcomes of anterior segment parameters after implanting an implantable collamer lens with a central hole (ICL V4c) in patients with myopia and determined the earliest follow-up time for detecting potential complications.

**Methods:**

Sixty-two patients were included, and the following parameters were measured at baseline (preoperative), 1 day, 1 week, and 1, 3, and 6 months after the operation: intraocular pressure (IOP), endothelial cell density (ECD), central anterior chamber depth (CACD), anterior chamber volume (ACV), nasal and temporal anterior chamber angle (n-ACA and t-ACA), horizontal corneal diameter (white-to-white, WTW), and axial length (AL). The vault was measured at each post-operative timepoint.

**Results:**

The postoperative IOP and ECD at the 6 months were both statistically similar to the baseline. The post-operative CACD and ACV were significantly less at all timepoints compared with the baseline (*P* < 0.001) and stayed stable from 1 day and 1 month after the operation, respectively. Postoperative n-ACA and t-ACA decreased significantly at 1 day and 1 week compared with the baseline (*P* < 0.001), while tended to stabilization at 1, 3, and 6 months. The vault kept decreasing significantly at 1 day, 1 week, and 1 month, but stayed stable at 3 and 6 months. The postoperative n-ACA and t-ACA positively correlated with the baseline ACA, CACD, and ACV.

**Conclusions:**

The anterior chamber parameters tended to stabilization early after the operation. Thus, it is essential to evaluate patients’ anterior segment status at earlier timepoints and prevent complications with prompt and non-invasive intervention.

## Background

The implantable collamer lens (ICL) is considered safe and efficient for myopic correction and is now widely accepted as an alternative treatment in patients at all refractive ranges [1–3]. However, conventional ICL interferes with the intraocular aqueous circulation and may result in potential complications, such as pupil block, elevated intraocular pressure (IOP), pigment dispersion syndrome, angle-closure glaucoma, metabolic cataract, and severe loss of endothelial cell density (ECD) [4].

The ICL V4c, a new kind of posterior ICL with a hole in the center of the lens, keeps the aqueous humor circulating naturally from the posterior to the anterior chamber. It largely maintains normal physiological function in the anterior segment and reduces the risk of aforementioned complications [5, 6]. It also showed long-term viability with stable visual acuity, intraocular pressure, and refraction [7–9]. However, a study [9] found that 6.8% patients developed asymptomatic anterior subcapsular cataract immediately after the implantation. There was also loss of ECD [8] which significantly correlated with anterior segment parameters such as anterior chamber volume (ACV), anterior chamber angle (ACA), the distance from the corneal endothelium to the central ICL, and vault. The ACA and vault are important parameters to evaluate intraocular safety and stabilization after ICL implantation [10, 11]. IOP could also increase due to residual ophthalmic viscosurgical device (OVD). Thus, it is essential to evaluate post-operative anterior segment parameters earlier. Timely observation of any changes in these parameters will help with prompt intervention, i.e., removing the ICL or conduct necessary procedure to remove residual OVD in earlier stages to prevent further invasive operations and reduce irreversible complications.

Nevertheless, there is still lack of evidence on the early outcomes of post-operative anterior segment parameters. Our study evaluated the post-operative central anterior chamber depth (CACD), ACV, ACA and other functional measurements relative to the preoperative baseline for timepoints up to 6 months, and determined the earliest follow-up time for detecting potential complications after ICL V4c implantation in patients with myopia.

## Methods

### Study design

This was a prospective, non-randomized, sequential case study. Each patient underwent ICL V4c (STAAR, Switzerland) implantation and adhered to scheduled follow-up visits in our hospital from February 2021 to May 2021.

The inclusion criteria were: aged ≥ 21 years; stable refractive error > 2 years, with < 0.50 D increase per year; CACD ≥ 2.8 mm; ECD ≥ 2000 cells/mm^2^; IOP 10 to 20 mmHg (1 mmHg = 0.133 kilopascal, kPa); and open ACA. The exclusion criteria were: history of any intraocular surgery, corneal abnormalities such as keratoconus, uveitis, glaucoma, retinal break, diabetes, or other autoimmune diseases; pregnant, breast-feeding, or with other conditions contraindicated for the surgery; sphere power < –18.00 D and cylinder power < –6.00 D; or unable to adhere to scheduled post-operative follow-up visits.

### Ethics approval

This study was approved by the Ethics Committee of Fudan University and was conducted in accordance with the principles of the Declaration of Helsinki. All the recruited patients signed the written informed consent forms for the surgery and any examinations after fully understanding the potential consequences.

### Preoperative examination

The preoperative routine examinations included the following: uncorrected visual acuity (UCVA); best corrected visual acuity (BCVA); non-mydriatic and mydriatic optometry; IOP; slit lamp microscopy; corneal topography of the central corneal thickness (CCT) and corneal curvature; anterior segment optical coherence tomography (AS-OCT); ultrasonic biological microscopy (UBM); ocular B-scan ultrasound; axial length (AL); dilated fundus; contact-free corneal endothelial microscopy; and the sulcus-to-sulcus.

IOP was measured with a non-contact tonometer (Canon Full Auto Tonometer TX-F; Canon, Tokyo, Japan). Corneal topography utilized a Pentacam (Oculus, Germany), and anterior segment optical coherence tomography was performed with a Carl Zeiss-Meditec Visante (Germany). UBM was via a model SW-3200 (Suoer Electric, China), and the ocular B-scan ultrasound source was from Tianjin Mida Medical Technology (MD2400S). AL was measured with an IOL Master (Zeiss, Germany). The contact-free corneal endothelial microscopy employed an SP-1P version 1.42 (Topcon, Japan).

### Surgical procedure

The same experienced physician performed the implantation of ICL V4c for all the patients. After ocular surface anesthesia, the conjunctival sac was rinsed, a routine disinfection towel was applied, and a skin protective membrane was affixed. The ICL V4c was placed in the pusher chamber head, adjusted to an appropriate position with lens tweezers, and the pusher chamber head was installed on the pusher.

A palpebral opener was placed, and a 3.0-mm-wide transparent corneal tunnel incision was made at the temporal limbus with a bayonet. ICL V4c was injected into the anterior chamber with a push injector, and sodium hyaluronate was injected into the anterior chamber. The 4 loops of ICL V4c were transferred into the ciliary sulcus with a positioning hook. The ICL V4c was adjusted to the appropriate position. The astigmatism type ICL V4c was adjusted according to the axial rotation diagram of the lens dispersion. The sodium hyaluronate was replaced with a rinse of balanced salt solution. After the operation, patients were treated with tobramycin and dexamethasone eye drops in the conjunctival sac.

Antibiotics, corticosteroids, and non-steroidal anti-inflammatory eye drops were routinely used to prevent post-operative inflammation.

### Postoperative follow-up

Slit lamp microscopy and IOP measurements were routinely performed at 2, 4, and 6 h after the surgery to observe the ocular condition of the patients. Patients with an IOP of 21 to 30 mmHg (1 mmHg = 0.133 kPa) 2 h after surgery were timely treated with carteolol eye drops. Patients with IOP > 30 mmHg were treated with appropriate drainage from the anterior chamber and the IOP was closely monitored. Patients were instructed to attend postoperative follow-up visits at 1 day, 1 week, and 1, 3, and 6 months, which included UCVA, computer optometry, IOP, slit lamp microscope examination, Pentacam, and Visante AS-OCT. UBM was performed at 1, 3, and 6 months to check whether the ICL loop was in the ciliary sulcus. Corneal endoscopy was performed 6 months after the surgery using non-contact specular microscopy (SP-1P version 1.42, Topcon, Japan) in accordance with the operation protocol. We chose the “CENTER” module for the fully automated measurement [12].

### Observation parameters

Preoperative manifest refractive spherical equivalent (SE), IOP, corneal curvature, horizontal corneal diameter (white-to-white, WTW), CACD, ACV and AL were routinely examined. Preoperative and postoperative ACA, CACD and postoperative vault were measured by Visante AS-OCT.

**Fig. 1:**
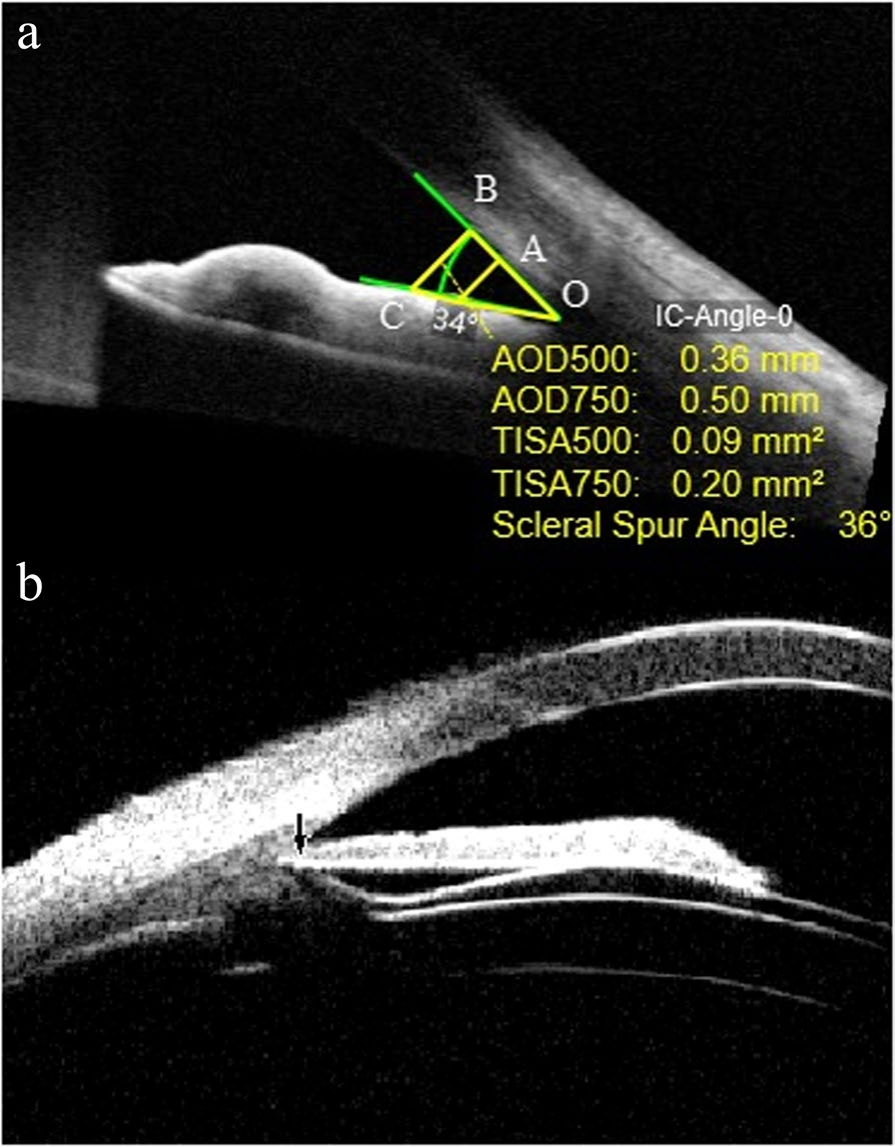
**a** Measurement method of anterior chamber angle (ACA) 1 week after implantable collamer lens with a central hole (ICL V4c) implantation. **b** The UBM image of ICL V4c posterior location

For ACA measurement, we set sclera spur as the starting point (marked as A point) and extended along the surface of corneal endothelial 500 microns (marked as point B). We marked a straight line perpendicular to the corneal endothelium to the iris from point B, and the intersection of the iris is point C. The apex of the angle recess was denoted as O. The angle formed by points B, C and O was ACA (Fig. 1a) We measured both nasal ACA (n-ACA) and temporal ACA (t-ACA).

Preoperative CACD was the distance between the posterior central surface of the cornea to the anterior surface of the lens. Postoperative CACD was the distance from the central posterior surface of the cornea to the anterior surface of ICL-V4c.

Postoperative vault was the distance from the center of the posterior surface of the ICL-V4c optical region to the anterior surface of the lens (Fig. 1b).

The same experienced ophthalmic technician measured all the parameters. The values of the CACD and vault reported herein are the respective means of the horizontal and vertical measurements that were taken 3 consecutive times.

### Statistical analysis

Data analysis was performed with SPSS 22.0 software. The Kolmogorov–Smirnov test was applied to test the normal distribution of the data. The anterior segment parameters conformed to a normal distribution and were shown as mean ± standard deviation. Repeated measures and variance tests were used to compare the changes in the anterior segment parameters at the different timepoints. The Bonferroni test was applied for comparisons of any 2 timepoints. The t-test was used to compare the nasal ACA (n-ACA) and temporal ACA (t-ACA) at the same timepoint. The correlation analysis was conducted by Pearson’s analysis. *P* < 0.05 was considered statistically significant.

## Results

### Preoperative and general postoperative characteristics

The study population comprised 62 sixty-two patients with myopia (62 right eyes), 32 men (32 eyes) and 30 women (30 eyes), aged 26.0 ± 4.6 years (range, 21 to 37 y). The preoperative characteristics are summarized in Table 1. All the surgeries were successful. In 3 eyes, the postoperative IOP at 2 h was 21 to 30 mmHg, which was controlled by carteolol eye drops until normal. There were 5 eyes with IOP larger than 30 mmHg and were immediately lowered to less than 15 mmHg via primary incision drainage. All the patients had IOP within the normal range at postoperative 4 to 6 h and all the follow-up visits. The UBM examination at 1, 3, and 6 months showed that the ICL V4c loops were in the ciliary sulcus without rotation or displacement. Slit lamp microscopy showed that the ICL V4c and lens surfaces were transparent. The UCVA of all the treated eyes reached or exceeded the preoperative BCVA. There was no significant difference in IOP or ECD between the baseline and 6 months after ICL V4c implantation (*P* > 0.05, Table 2). There were no complications such as cataract, glaucoma, or ICL deviation.

**Table 1:** Summary of the preoperative characteristics

	Mean ± SD
Age, y	26.0 ± 4.6
Spherical equivalent, diopter	–7.56 ± 2.55
Average corneal horizontal curvature, degree	42.89 ± 1.47
White-to-white horizontal corneal diameter, mm	11.64 ± 0.37
Central anterior chamber depth, mm	3.19 ± 0.21
Anterior chamber volume, mm^3^	210.31 ± 27.75
Axial length, mm	26.64 ± 0.93

*SD* Standard deviation

**Table 2:** Anterior segment parameters measurement at the baseline and after implantable collamer lens with a central hole (ICL V4c) implantation

		IOP, mmHg	ECD, cells/mm^2^	CACD, mm	ACV, mm^3^
Baseline		15.97 ± 2.13	2989.30 ± 140.78	3.19 ± 0.21	210.30 ± 27.7
Postoperative	1 day	16.02 ± 1.58	—	3.01 ± 0.23 ^a^	104.31 ± 21.20 ^a^
	1 week	16.13 ± 1.49	—	3.00 ± 0.21 ^a^	109.35 ± 22.17 ^a,b^
	1 month	16.18 ± 1.35	—	3.01 ± 0.20 ^a^	110.71 ± 21.52 ^a,b^
	3 months	15.97 ± 1.29	—	3.02 ± 0.19 ^a^	111.77 ± 21.08 ^a,b^
	6 months	16.16 ± 1.08	2958.44 ± 126.78	3.03 ± 0.19 ^a^	111.04 ± 19.74 ^a,b^
*F*		0.875	1.23	11.10	288.38
^c^*P* value		0.504	0.204	** < 0.001**	** < 0.001**

*ACV* Anterior chamber volume, *CACD* Central anterior chamber depth, *ECD* Endothelial cell density, *IOP* Intraocular pressure

^a^ Compared with preoperative (baseline), all *P* < 0.0001 and there were no significant differences at different post-operative timepoints

^b^ compared with 1 day after ICL V4c implantation, all *P* < 0.0001 and there were no significant differences at 1 week or other post-operative timepoints

^c^ multivariate test

### Anterior segment parameter analysis

#### IOP

The baseline IOP was 15.97 ± 2.13 mmHg. IOP at 1 day, 1 week, and 1, 3, and 6 months after ICL V4c implantation were 16.02 ± 1.58, 16.13 ± 1.49, 16.18 ± 1.35, 15.97 ± 1.29, and 16.16 ± 1.08 mmHg, respectively. The difference was not statistically significant (*F* = 0.875, *P* = 0.504, Table 2).

#### ECD

The ECD showed no significant difference between the baseline (2989.30 ± 140.78 cells/mm^2^) and 6-month (2958.44 ± 126.78 cells/ mm^2^) measurements (*F* = 1.23, *P* = 0.204, Table 2).

#### CACD

The postoperative CACD measurements at 1 day, 1 week, and 1, 3, and 6 months were 3.01 ± 0.23, 3.00 ± 0.21, 3.01 ± 0.20, 3.02 ± 0.19, and 3.03 ± 0.19 mm, respectively, which were all significantly less than that of the baseline CACD (3.19 ± 0.21 mm; *F* = 11.10, *P* < 0.001, Table 2). However, postoperative CACD stayed stable 1 day after the operation as there were no significant differences among the postoperative timepoints at 1 day, 1 week, and 1, 3, and 6 months (*P* > 0.05, Table 2).

#### ACV

The postoperative ACV measurements at 1 day, 1 week, and 1, 3, and 6 months were 104.31 ± 21.20, 109.35 ± 22.17, 110.71 ± 21.52, 111.77 ± 21.08, and 111.04 ± 19.74 mm^3^, respectively, which were significantly lower than the baseline ACV of 210.30 ± 27.7 mm^3^ (*F* = 288.38, *P* < 0.001, Table 2). Although it significantly increased at one week compared with one day (*P* < 0.001), it stayed stable at 1, 3, and 6 months. (*P* > 0.05, Table 2).

#### Vault

The postoperative vault continued to reduce significantly at 1 day, 1 week, and 1, 3, and 6 months, with vault measurements of 665.32 ± 184.03 µm, 597.42 ± 166.33, 574.68 ± 159.43, 570.65 ± 150.52 µm, and 567.90 ± 152.32 µm, respectively (*F* = 52.10, *P* < 0.001). However, it stayed stable at 3 months and 6 months, showing no significant difference when compared with the measurement at 1 month.

#### ACA

The n-ACA and t-ACA were comparable at baseline (t = 1.17, *P* = 0.25; Table 3), and post-operative timepoints 1, 3, and 6 months (t = 0.16, *P* = 0.86; t = 1.91, *P* = 0.06; t = 0.47, *P* = 0.64, respectively). The t-ACA was significantly wider than the n-ACA at the post-operative timepoints 1 day (t = 8.70, *P* < 0.001) and 1 week (t = 4.66, *P* < 0.001).

**Table 3:** The nasal and temporal anterior chamber angle at the baseline and after ICL V4c implantation

		n-ACA	t-ACA	t	P
Baseline		40.60 ± 2.47	41.16 ± 4.60	1.17	0.25
Postoperative	1 day	22.68 ± 2.86	23.14 ± 2.72	8.70	< 0.001
	1 week	23.16 ± 3.17 *	23.38 ± 3.11	4.66	< 0.001
	1 month	23.10 ± 3.08 *	23.18 ± 2.94	0.16	0.86
	3 months	23.11 ± 3.07 *	23.17 ± 3.00	1.91	0.06
	6 months	23.14 ± 3.09 *	23.12 ± 3.02	0.47	0.64
*F*		1279	239.2		
*P*		< 0.001	< 0.001		

*n-ACA* Nasal anterior chamber angle, *t-ACA* Temporal anterior chamber angle

^*^ Compared with 1 day after ICL V4c implantation, *P* < 0.05

**Fig. 2:**
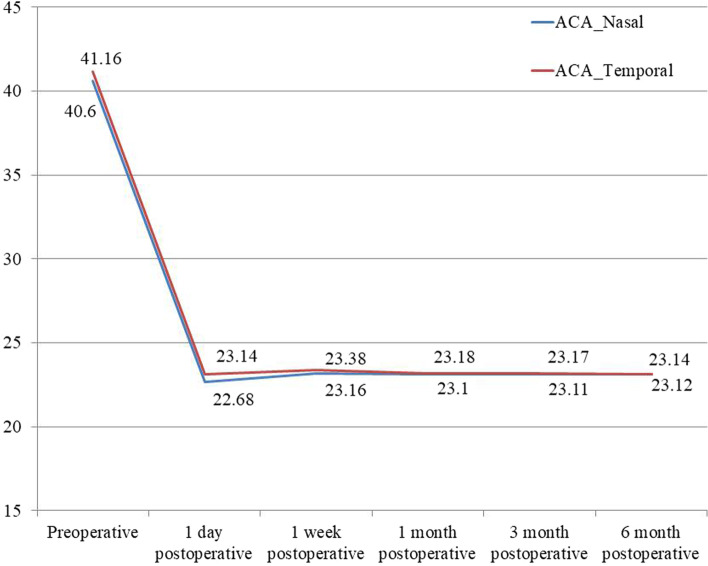
The nasal and temporal anterior chamber angle (n-ACA and t-ACA) changes over time after implantable collamer lens with a central hole (ICL V4c) implantation and anterior segment parameters

Compared with the baseline measurement, the n-ACA and t-ACA were lower by 17.9° (44.1%) and 18.02° (43.78%), respectively, 1 day after the surgery. Although all the postoperative n-ACA and t-ACA measurements were significantly lower compared with the respective baseline measurements (*F* = 1279, *P* < 0.001; *F* = 239.2, < 0.001, Table 3), the postoperative measurements at 1, 3, and 6 months were similar. Figure 2 also showed that the measurements stayed stable 1 month after the operation.

The n-ACA measurements were positively associated with the preoperative n-ACA, CACD, and ACV at 1 day, 1 week, and 1, 3, and 6 months after the implantation, but no association with baseline AL and WTW, and postoperative vault. (Tables 4) The t-ACA measurements were also positively associated with the preoperative t-ACA, CACD, and ACV at 1 day, 1 week, and 1, 3, and 6 months after the implantation, but no association with baseline AL and WTW, and postoperative vault (Table 4).

**Table 4:** Correlation analysis of a) the post-operative nasal anterior chamber angle (n-ACA) and the anterior segment parameters, and b) the post-operative temporal anterior chamber angle (t-ACA) and other anterior segment parameters

(a) The correlation of n-ACA and the anterior segment parameters at different time points										
	1 day		1 week		1 month		3 months		6 months	
	*r*	*P*	*r*	*P*	*r*	*P*	*r*	*P*	*r*	*P*
AL	–0.074	0.57	–0.08	0.530	–0.087	0.5	–0.092	0.475	–0.093	0.472
WTW	–0.043	0.821	–0.027	0.834	–0.061	0.637	–0.068	0.597	–0.065	0.614
ACD (baseline)	0.714	** < 0.001**	0.715	** < 0.001**	0.711	** < 0.001**	0.705	** < 0.001**	0.706	** < 0.001**
ACV (baseline)	0.764	** < 0.001**	0.738	** < 0.001**	0.737	** < 0.001**	0.736	** < 0.001**	0.739	** < 0.001**
n-ACA (baseline)	0.795	** < 0.001**	0.748	** < 0.001**	0.762	** < 0.001**	0.756	** < 0.001**	0.761	** < 0.001**
Vault	–0.015	0.906	–0.013	0.918	–0.052	0.687	–0.042	0.748	–0.039	0.765
b The correlation of t-ACA and the anterior segment parameters at different time points										
	1 day		1 week		1 month		3 months		6 months	
	*r*	*P*	*r*	*P*	*r*	*P*	*r*	*P*	*r*	*P*
AL	–0.054	0.675	–0.059	0.647	–0.087	0.5	–0.096	0.459	–0.087	0.499
WTW	–0.037	0.776	–0.019	0.886	–0.061	0.637	–0.077	0.554	–0.085	0.511
ACD (baseline)	0.709	** < 0.001**	0.724	** < 0.001**	0.711	** < 0.001**	0.699	** < 0.001**	0.694	** < 0.001**
ACV (baseline)	0.765	** < 0.001**	0.759	** < 0.001**	0.737	** < 0.001**	0.734	** < 0.001**	0.733	** < 0.001**
t-ACA (baseline)	0.475	** < 0.001**	0.432	** < 0.001**	0.459	** < 0.001**	0.450	** < 0.001**	0.457	** < 0.001**
Vault	0.013	0.918	–0.011	0.935	–0.052	0.687	–0.044	0.734	–0.055	0.672

*ACD* Anterior chamber depth, *ACV* Anterior chamber volume, *AL* Axial length, *n-ACA* Nasal anterior chamber angle, *t-ACA* Temporal anterior chamber angle, *WTW* White-to-white horizontal corneal diameter

## Discussion

To the best of our knowledge, this study is the first to focus primarily on early outcomes of anterior segment parameters from 1 day to 6 months after ICL V4c implantation. The results showed that the post-operative IOP and ECD at 6 months were comparable to the baseline measurements. The CACD and ACV were significantly lower at 1 day and 1 week, and tended toward stabilization 1 week after the implantation. Compared with the vault measurement at 1 day, the values were significantly lower at 1 week and 1 month, but stayed stable at 1, 3, and 6 months. Postoperative n-ACA and t-ACA were significantly lower at 1 day and 1 week compared with the baseline, but tended to stabilization from 1, 3, and 6 months, and the t-ACA was slightly wider than the n-ACA. The postoperative n-ACA and t-ACA positively correlated with the baseline ACA, CACD, and ACV at all timepoints, but not with baseline WTW and AL, or postoperative vault.

Measuring the posterior vault is essential to evaluate the risk of complications such as cataract and glaucoma after ICL implantation [8, 13]. A low vault resulting in a closed distance between the ICL and the anterior capsule of the patient’s own lens, may lead to anterior subcapsular cataract [2, 14]. A high vault increases the friction between the posterior surface of the iris and the surface of the ICL, leading to the loss of iris pigment [15]. In addition, it may lead to a shallow anterior chamber affecting aqueous humor drainage, secondary angle closure, and pigment dispersion syndrome [15]. Thus, it important to ensure that the vault is in a relatively safe range (i.e., ≥ 150 µm). It is reported that the ideal vault ranges from 250 to 750 µm, and a postoperative vault ranging from 750 to 1000 µm is also acceptable [16]. Our results showed that at 6 months after the implantation, the vault ranged from 270 to 900 µm, with an average of 567.9 ± 153.67 µm, with 90.3% (56 of 62 eyes) in the ideal vault range, and 9.7% (6/62) in the range of 750 to 1000 µm. The vault at 1 week after implantation decreased significantly relative to the first day. The vault at 1 month was also significantly less than at 1 week but stayed stable afterwards. As the vault decreases with the aqueous humor circulation and the metabolism of sodium hyaluronate, measurements at earlier follow-up visits (i.e., before 1 month) can help with timely evaluation of any abnormalities and potentially reduce further complications. Specifically, the evaluation of the relative stability of the vault within 1 week after ICL V4c implantation is particularly crucial to guide the early replacement with more appropriate ICL if there is a risk of cataract.

It is also important to evaluate the ACA width after the ICL implantation, because an extremely narrow ACA may lead to angle-closure glaucoma [17, 18]. An ACA at level 2 (20°) could significantly increase the possibilities of peripheral-anterior adhesion and peripheral-angle closure [19]. Most previous studies did not measure the ACA in ICL-implanted patients at 1 week, but waited until 1 month, 3 months, or even longer, and they did not analyze n-ACA and t-ACA separately, which may increase the risk of unnoticed early complications. In the present study, we observed the ACA promptly and closely, and there was no significant difference between the baseline n-ACA and t-ACA, but the t-ACA was significantly wider than the n-ACA at 1 day and at 1 week after the surgery.

This might be related to the temporal location of the incision and operation. Intraoperative stimulation of the temporal iris and the residue of a small amount of sodium hyaluronate on the incision side may cause temporary differences between the t-ACA and n-ACA. However, the differences at 1 week were significantly lower than at 1 day. This may be due to the incomplete recovery of intraoperative stimulation of the iris near the incision and the healing of the incision at the earlier follow-up visits [6]. The differences became insignificant in the subsequent follow-up visits at 1, 3, and 6 months after surgery, indicating that the n-ACA and t-ACA were stable after 1 week when the incision healed and intraocular structure stabilized. Thus, it is important to observe changes of the ACA as early as 1 week to detect any abnormal signs such as convex iris convex, angle adhesion, and pigment dispersion and prevent irreversible complications.

Our results are consistent with a previous report [20], showing that the post-operative CACD and ACV were all significantly lower than the baseline and remained stable at 1 week and thereafter. The peripheral anterior chamber depth also has good sensitivity for detecting the risk of ACA closure [17, 21]. The present correlation analysis showed that the n-ACA and t-ACA at 1 day, 1 week, and 1, 3, and 6 months after the implantation were significantly and positively associated with the baseline ACA, CACD, and ACV. Therefore, for patients with a narrow ACA (e.g., less than 20 degree), shallow anterior chamber, or small ACV at the baseline, caution is appropriate when choosing ICL V4c implantation as the treatment, as patients may be more vulnerable to postoperative complications [17, 22].

This study has several strengths. First, we included patients with myopia with a large range of SE (–3.00 to –11.50) to evaluate the outcomes of ICL V4c implantation for different disease severities. Second, the same technician used Visante AS-OCT under the same light conditions for baseline and postoperative examinations to prevent the potential effects of pupil size changes due to different light intensity. Third, we provided short-term vault document which could help with the early evaluation of ICL position. The study is also limited, in that we did not compare the anterior segment parameters of both eyes of the same patient and there was potential for an intra-eye effect. Second, the patients were all younger than 40 years old and the follow-up time was relatively short (6 months). The evaluation may not be comprehensive enough to reflect the progression of some complications such as cataract. Third, we did not include other potentially important factors into the analysis, such as the change of postoperative aqueous outflow, the peripheral-angle closure, the patient's age, and the influence of the ICL size on the measurement. Studies found that post-operative ACA is related to the diameter of the implanted ICL [23] and the larger lens size caused greater changes on the ACA [17]. Fourth, due to the small sample size, our study could not prove the clinically significant differences of those measurements with statistically significant differences. Thus, future studies with a larger patient sample, variant ICL sizes, and longer follow-up time are warranted for a more comprehensive investigation.

## Conclusion

In conclusion, ICL V4c appeared viable during the 6 months’ postoperative follow-up and the anterior chamber parameters tended to stability early after the implantation. Thus, it is essential to evaluate patients’ anterior segment status at earlier timepoints (e.g., 1 week and 1 month) and prevent complications with prompt and non-invasive intervention.

## Data Availability

The datasets generated and analysed during the current study are not publicly available due to limitations of ethical approval involving the patient data and anonymity but are available from the corresponding author on reasonable request.

## Abbreviations

ICL: Implantable collamer lens
IOP: Intraocular pressure
ECD: Endothelial cell density
ACV: Anterior chamber volume
ACA: Anterior chamber angle
OVD: Ophthalmic viscosurgical device
CACD: Central anterior chamber depth
UCVA: Uncorrected visual acuity
BCVA: Best corrected visual acuity
CCT: Central corneal thickness
AS-OCT: Anterior segment optical coherence tomography
UBM: Ultrasonic biological microscopy
AL: Axial length
